# 14,15-Dihydroxyeicosatrienoic acid, a soluble epoxide hydrolase metabolite in blood, is a predictor of anthracycline-induced cardiotoxicity – a hypothesis generating study

**DOI:** 10.1186/s40959-023-00198-7

**Published:** 2023-12-15

**Authors:** Julia Matzenbacher dos Santos, Aby Joiakim, David A. Putt, Marielle Scherrer-Crosbie, Hyesook Kim

**Affiliations:** 1 Detroit R&D, Inc., 2727 2nd Ave, Suite 4113, Detroit, MI 48201 USA; 2 Department of Health Promotion and Development, School of Nursing, University of Pittsburgh, Pittsburgh, PA 15261 USA; 3 Cardiac Ultrasound Laboratory, Hospital of the University of Pennsylvania, Philadelphia, PA 19104 USA; 4 Institute of Environmental Health Sciences, Wayne State University, Detroit, MI 48202 USA

**Keywords:** Cardiotoxicity, 14,15-DHET, Early blood biomarker, Anthracyclines, Doxorubicin, Soluble epoxide hydrolase, Chemotherapy, Breast cancer, Cardio-oncology, LVEF

## Abstract

**Background:**

Early identification of patients susceptible to chemotherapy-induced cardiotoxicity could lead to targeted treatment to reduce cardiac dysfunction. Rats treated with doxorubicin (DOX), a chemotherapeutic agent, have increased cardiac expression of 14,15-dihydroxyeicosatrienoic acid (14,15-DHET), a bioactive lipid implicated in hypertension and coronary artery disease. However, the utility of 14,15-DHET as plasma biomarkers was not defined. The aim of this study is to investigate if levels of 14,15-DHET are an early blood biomarker to predict the subsequent occurrence of cardiotoxicity in cancer patients after chemotherapy.

**Methods:**

H9c2 rat cardiomyocytes were treated with DOX (1 μM) for 2 h and levels of 14,15-DHET in cell media was quantified at 2, 6 or 24 h in media after DOX treatment. Similarly, female Sprague–Dawley rats were treated with DOX for two weeks and levels of 14,15-DHET was assessed in plasma at 48 h and 2 weeks after DOX treatment. Changes in brain natriuretic peptide (BNP) mRNA, an early cardiac hypertrophy process, were determined in the H9c2 cells and rat cardiac tissue. Results were confirmed in human subjects by assessment of levels of 14,15-DHET in plasma of breast cancer patients before and after DOX treatment and left ventricular ejection fraction (LVEF), a clinical marker of cardiotoxicity.

**Results:**

Levels of 14,15-DHET in cell media and rat plasma increased ~ 3-fold and was accompanied with increase in BNP mRNA in H9c2 cells and rat cardiac tissue after DOX treatment. In matched plasma samples from breast cancer patients, levels of 14,15-DHET were increased in patients that developed cardiotoxicity at 3 months before occurrence of LVEF decrease.

**Conclusions:**

Together, these results indicate that levels of 14,15-DHET are elevated prior to major changes in cardiac structure and function after exposure to anthracyclines. Increased levels of 14,15-DHET in plasma may be an important clinical biomarker for early detection of anthracycline-induced cardiotoxicity in cancer patients.

## Background

Approximately 610,000 cancer-related deaths will occur in 2023 in the United States [1]. Highly effective chemotherapy drugs such as doxorubicin (DOX) and trastuzumab are widely used to treat cancers including breast, ovarian and lung cancers [2]. However, these treatments can induce left ventricular dysfunction and subsequent heart failure [3].

Left ventricular ejection fraction (LVEF) is not sensitive in the detection of early cardiotoxicity. Whereas two-dimensional speckle tracking echocardiography (2D STE) which measures left ventricular global longitudinal strain (LVGLS) can detect subclinical dysfunction [4], facile and reliable blood biomarkers for early detection of chemotherapy-induced cardiotoxicity are needed to reduce incidents of ventricular dysfunction or heart failure.

According to the new guideline from the European Society of Cardiology Cardio-oncology elevation of Troponin (TN) and brain natriuretic peptide (BNP) are blood biomarkers that should be monitored to predict cardiomyocyte injury and ventricular remodeling during chemotherapy treatment [5]. However, these biomarkers do not offer an opportunity for targeted treatment.

In addition, recent studies have tested if circulating microRNAs (miRNAs) can be used as a biomarker for DOX-induced cardiotoxicity in cancer patients [6, 7]. Several potential miRNA candidates were identified, but the lack in consistence among studies is so far a major shortcoming that impede the use of miRNA biomarkers for DOX-induced cardiotoxicity.

14,15-Dihydroxyeicosatrienoic acid (14,15-DHET)- and 20-hydroxyeicosatetraenoic acid (20-HETE)-formation activities of soluble epoxide hydrolase (sEH) and cytochrome P450 (CYP) 4A/4F, respectively, increased in the heart of rats 24 h after they had been treated with a single dose of DOX (15 mg/kg) [8] and sEH and CYP4A/4F could be targets suitable for intervention. However, it is not known yet whether these fatty acids are early blood biomarkers of DOX-induced cardiotoxicity. The aim of this study is to investigate if levels of 14,15-DHET are an early biomarker that can be used to predict the subsequent occurrence of cardiotoxicity in cancer patients after anthracycline treatment.

## Methods

### Materials

A rat myocardium H9c2 cell line was obtained from American Type Culture Collection (ATCC, Manassas, VA). 14,15-DHET and 20-HETE ELISA kits and 14,15-epoxyeicosatrienoic acid (EET) were obtained from Detroit R&D, Inc. (Detroit, MI). DOX, 3-(4,5-dimethylthiazol-2-yl)-2,5-diphenyl tetrazolium bromide (MTT) and Dulbecco’s modified Eagle’s medium (DMEM) supplemented with 0.45% glucose were purchased from Sigma-Aldrich (St. Louis, MO), High-Capacity DNA Reverse Transcription kit was from Applied Biosystem (Foster City, CA), SYBR green master mix was from ThermoFisher Scientific (Pittsburg, PA) and BNP and β-actin forward and reverse primers were from Integrated DNA Technology (Coralville, IA).

### In-vitro assay

**Fig. 1:**
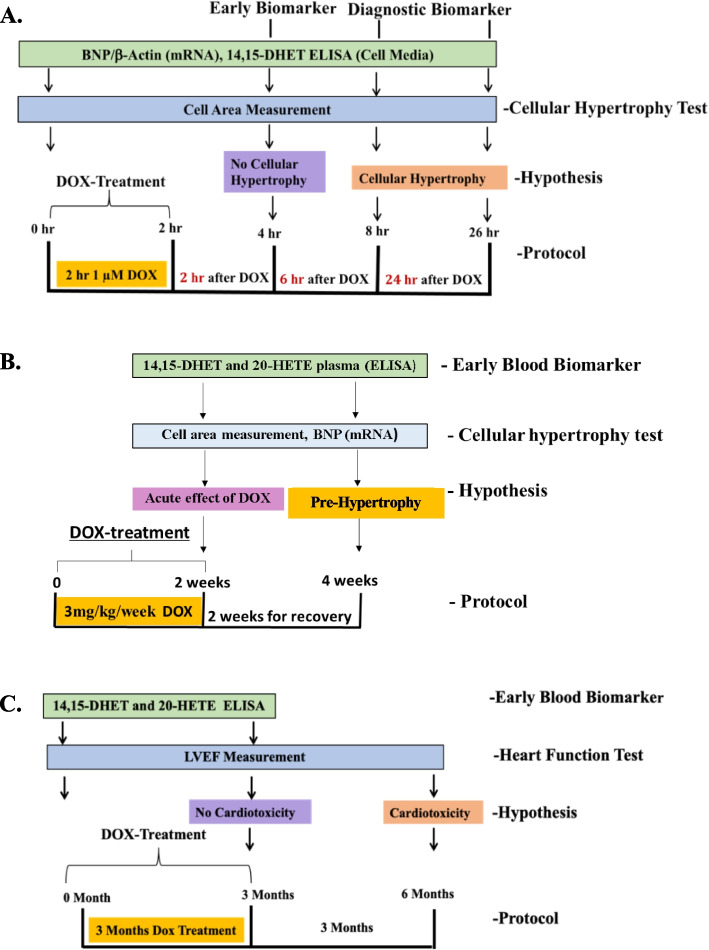
Experimental design of early blood biomarker study for doxorubicin (DOX)-induced cardiotoxicity. The experiments are planned for in-vitro and in-vivo in rodents and breast cancer patients*. ***A** In-vitro verification of early and diagnosis biomarkers of doxorubicin (DOX)-induced cardiotoxicity, 14,15-DHET, using culture media obtained from H9c2 rat cardiomyocytes. **B** Confirmation of the in-vitro results using female rates treated with DOX (3 mg/kg). **C** Breast cancer human plasma analyses prior and after DOX treatment. Cardiotoxicity is defined as an asymptomatic reduction of LVEF of ≥ 10% to < 55% [9]

Rat myocardium H9c2 cells (ATCC) were grown in DMEM supplemented with 0.45% glucose. When cells reached 80–90% confluency, media was replaced with media containing DMSO (0.01%) for controls or 1 µM DOX (Sigma-Aldrich) dissolved in DMSO (0.01%). Cells were treated for 2 h, washed and kept for 2, 6 or 24 h in media without DOX (Fig. 1A). Cell culture assays were performed in 2–3 replicates using 4–6 plates using 30 mm culture dishes for each replication.

### In-vivo assay

Female Sprague–Dawley rats (8 weeks old) purchased from Charles River (Ashland, OH) were kept for 7 days in quarantine and, then they received two intravenous injections of either 3 mg/kg body weight of DOX (DOX-treated, *n* = 12) or a similar volume of saline (Control, *n* = 12). The injections (DOX or saline) were carried out on the first day and the seventh day of the experimental protocol (one week after the first injection). Half of the animals in each group (*n* = 6 per group) were sacrificed at 48 h after the second injection. The other half of the animals was sacrificed 2 weeks after the second injection (Fig. 1B). Blood and heart tissue were collected.

### Human samples

Ten coded plasma samples obtained from 5 HER2-positive breast cancer patients were provided by Dr. Scherrer-Crosbie from Massachusetts General Hospital and Harvard Medical School (Boston, MA). This is a secondary use for the specimens. Use of the coded plasma samples for this study (NHLBI SBIR contract N261201600028C) was determined by the Wayne State University (Detroit, MI) Human Participant Research (HPR) Determination (#2016 78). Patients were fasted for at least 8 h prior to the sample collection. Tubes containing ethylenediaminetetraacetic acid (EDTA) were used for blood collection to prevent coagulation. After plasma isolation, samples were stored at –80 °C until the time of assay.

Patients older than 18 years old diagnosed with HER2-overexpressing breast cancer were eligible. Patients with LVEF levels lower than 50% at the baseline were excluded from this experiment. The baseline LVEF levels for the 5 patients were between 64% and 72% (mean LVEF, 69%). Patients were treated with DOX (60 mg/m^2^) and cyclophosphamide (600 mg/m^2^) for 3 months (every 3 weeks for 4 cycles). At 3 months, paclitaxel (80 mg/m^2^) was administered concurrently with trastuzumab every week. LVEF was measured before and after three and six months after the beginning of 3 months of anthracycline treatment as previously described [3] (Fig. 1C).

Briefly, digital 2D echocardiograms were analyzed using the EchoPAC workstation (GE Healthcare) in a core laboratory. Left ventricular end-diastolic and end-systolic volumes were measured using Simpson’s method of discs in the apical 4- and 2-chamber views [10]. The reader was blinded to the clinical or biomarker data of the patients. Cancer therapy-related cardiac dysfunction (CTRCD) or chemotherapy-associated cardiotoxicity is defined as a reduction in LVEF of ≥ 5% to < 55% with symptoms of heart failure or an asymptomatic reduction of LVEF of ≥ 10% to < 55% [9]. The patients included in this experiment were asymptomatic.

### Soluble epoxide hydrolase (sEH) activity assay

Activity assay of sEH was performed by incubating H9c2 cells with either 1 μM or 5 μM of the sEH substrate, 14,15-EET. After 1 h of incubation, media was collected and 14,15-DHET was measured by 14,15-DHET ELISA kit (Detroit R&D, Inc.).

### Cell viability

Cell viability after DOX treatment was assessed with MTT (Sigma-Aldrich) as previously described [11].

### Cardiomyocyte hypertrophy

H9c2 size was assessed by microscopy. Cells were fixed and stained with Mayer’s hematoxylin reagent and eosin Y (ScyTek). Images of the cells were taken using a Zeiss Axiovert 200 microscope with RT Insight Camera and SPOT Advanced Imaging software at 40X magnification. Cellular areas were obtained using Image J software.

### mRNA expression

For mRNA expression assessment, RNA from cells and rat heart was isolated by TRIzol/chloroform method. Briefly, pellets were incubated with 1 ml of TRIzol reagent for 5 min (Invitrogen, Carlsbad, CA), 0.2 ml of chloroform was added to the solution, mixed and centrifuged for 15 min (12,000 xg). RNA from the aqueous phase was collected, mixed with 0.5 ml of 2-propanol (Sigma-Aldrich), centrifuged for 10 min (12,000 xg) and pellets were washed with 75% ethanol. Pellets were suspended with nuclease-free water and cDNA was synthesized using High-Capacity DNA Reverse Transcription kit (Applied Biosystem, Foster City, CA). Real time qPCR was performed using SYBR green master mix and rat specific BNP forward primer, 5’- CAGAAGCTGCTGGAGCTGATAAG-3’, and reverse primer, 5’-TGTAGGGCCTTGGTCCTTTG-3’; myeloperoxidase (MPO) forwards primer 5’-TTTGACAGCCTGCACGATGA-3’, reverse primer 5’- GTCCCCTGCCAGAAAACAAG- 3’. Results were normalized by β-actin mRNA expression using forward primer, 5’-CCAGATCATGTTTGAGACCTTCAA-3’, and reverse primer, 5’-GTGGTACGACCAGAGGCATACA-3’.

### 14,15-DHET and 20-HETE immunoassays

LVEF information and cardiotoxicity status of each coded patient was not revealed until after biomarker analyses were performed. ELISA was performed using coded plasma samples at Detroit R&D in a double-blind fashion. Ethyl acetate extraction of the plasma samples (35 µl) was carried out and levels of the fatty acids, in triplicate, were assessed using 14,15-DHET and 20-HETE ELISA kits from Detroit R&D, Inc. The extraction protocol was in accordance with the manufacturer’s instructions, which was previously used by others [12, 13]. Levels of 14,15-DHET and 20-HETE were also assessed in rat plasma after extraction with ethyl acetate. For cell study, levels of 14,15-DHET were measured in cell media after ethyl acetate extraction. The same immunoassays, 14,15-DHET and 20-HETE ELISA kits (Detroit R&D, Inc), were used for human and rat plasma, and cell media analyses.

### Statistical analyses

Results were analyzed using the SPSS software IBM (Armonk, NY) and Excel software Microsoft (Seattle, WA). Normality test was performed using Shapiro–Wilk test. Paired Student t-tests were used to analyze human data using triplicate values obtained by ELISA for each patient after chemotherapy compared to triplicate values obtained before chemotherapy. Pearson correlation was used to test the relationship between changes in 14,15-DHET and LVEF. Results of cell studies were analyzed using unpaired student t-tests. If the data had presented any abnormal distribution, non-parametric tests would have been used for statistical analyses. Statistical significance was set at *p* < 0.05.

## Results

**Fig. 2:**
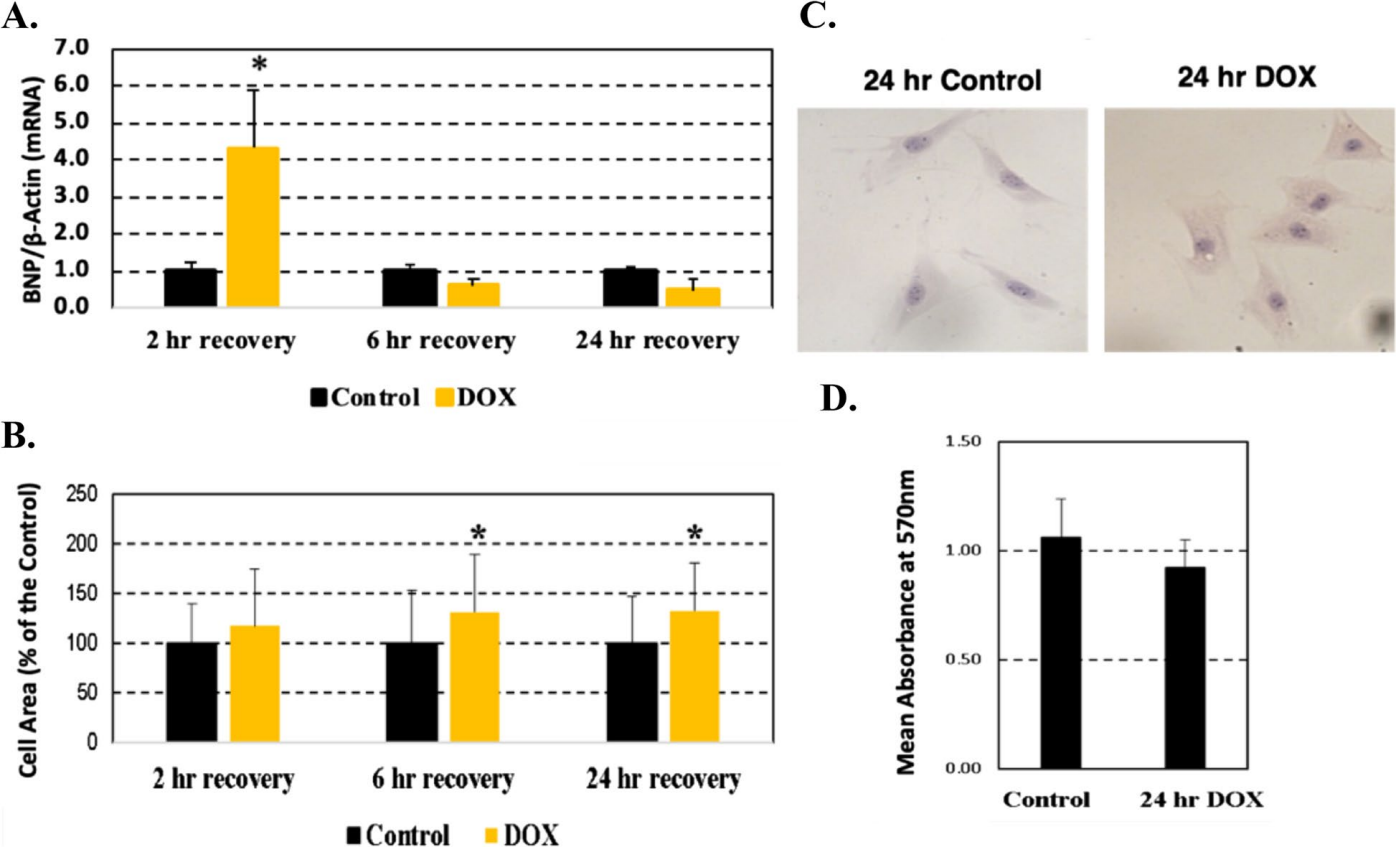
DOX treatment induced BNP expression and cell hypertrophy in H9c2 rat cardiomyocytes. Cells were collected at 2 h, 6 h and 24 h with 2 h pre-treatment of DMSO (0.01%) for controls or 1 µM DOX (Sigma) dissolved in DMSO (0.01%). **A** Levels of BNP mRNA assessed with rat specific BNP primers and normalized by β-actin mRNA in duplicate. **B** Cell area measured using light microscopy and Image J software. **C** Representative cell images obtained by light microscopy. **D** Cell viability measured by the MTT assay**.** Results were expressed as mean ± SD. *p < 0.05 compared to cells not treated with DOX using an independent T-test. The assays were performed in three replications using 4–6 plating for each replication

**Fig. 3:**
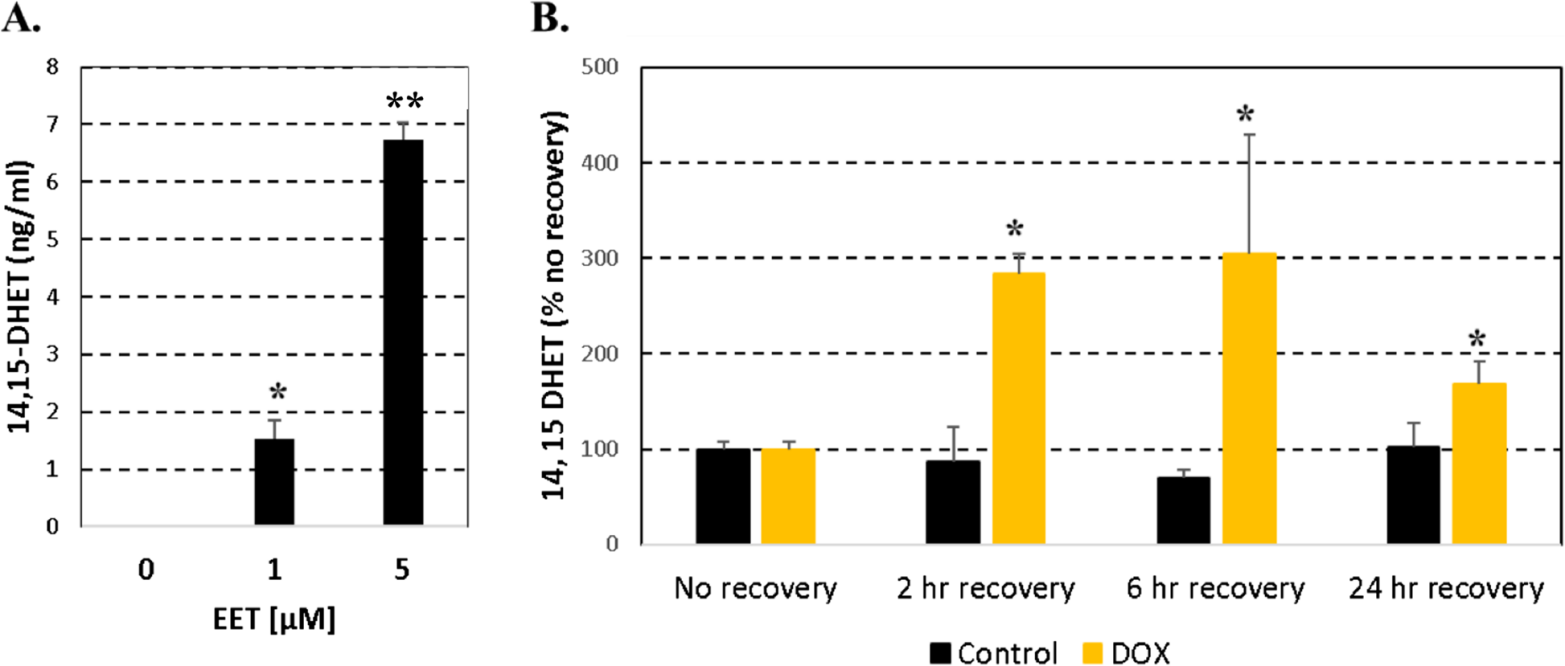
DOX treatment induced 14,15-DHET expression in culture media of H9c2 rat cardiomyocytes. Cells were collected at 2 h, 6 h and 24 h with 2 h pre-treatment of DMSO (0.01%) (Control) or 1 µM DOX dissolved in DMSO (0.01%). **A** Levels of 14,15-DHET in culture media of H9c2 cardiomyocytes treated for 60 min with 0, 1 and 5 µM 14,15-EET. **B** Levels of 14,15-DHET in cell media measured by ELISA. Results were expressed as mean ± SD. *p < 0.05 and **p < 0.01 compared to cells not treated with 14,15-EET or DOX using an independent T-test. The assays were performed in two replications using 4 plating for each replication

An in-vitro test was performed using H9c2 rat cardiomyocytes to determine whether 14,15-DHET secreted in culture media is an early biomarker of DOX-dependent cardiotoxicity (Fig. 1A). DOX-induced cellular hypertrophy of the cardiomyocytes was assessed by measurement of cell area. Expression of BNP mRNA normalized by β-actin mRNA expression increased several folds at 2 h after 2 h of 1 µM DOX treatment (Fig. 2A) when no change in cell area was detected (Fig. 2B). BNP expression levels decreased at 6 h and 24 h after 2 h of 1 µM DOX treatment (Fig. 2A) when cellular hypertrophy was detected (Fig. 2B and C). Whereas no changes were observed at 2 h after 2 h treatment with 1 µM DOX, cell area increased at 6 h and 24 h of recovery period (Fig. 2B). An increase of cellular hypertrophy measured at 24 h of recovery was observed with minimal changes in cellular viability (Fig. 2D). Further analyses using DOX-treated H9c2 rat cardiomyocytes (1 µM DOX for 2 h) confirmed a role for sEH since 14,15-DHET increased as its substrate 14,15-EET increased (Fig. 3A). Levels of 14,15-DHET increased in cell media after 2 h of recovery, prior to detection of cellular hypertrophy at 6 h and 24 h of recovery (Fig. 3B).

**Fig. 4:**
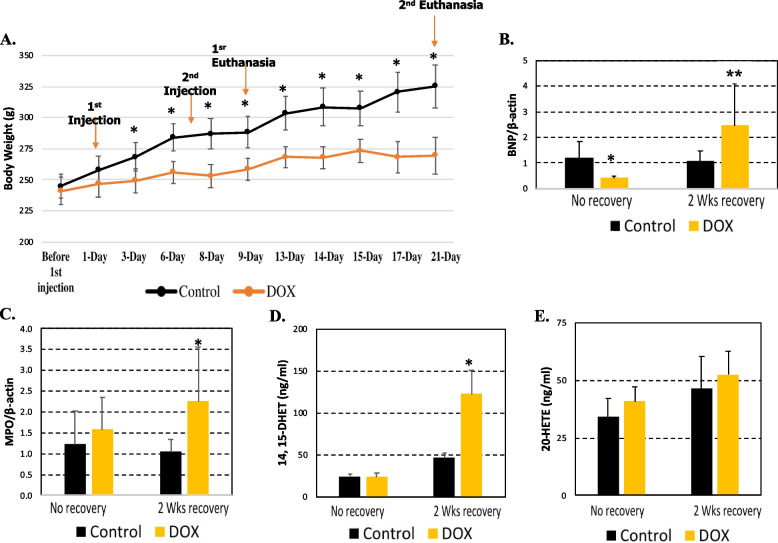
Plasma levels of 14,15-DHET in rats following DOX treatment. Dox (3 mg/kg/week) was injected in female Sprague–Dawley rats (*n* = 12) whereas the same volume of saline was injected in control rats. **A** Body weight and percent of weight gain was assessed everyday over the course of the experiment. Rats were euthanized 48 h (no recovery) or after 2 weeks (2 wks recovery) of injections with saline (control) or 3 mg/kg/week DOX. Hearts were collected and RNA was isolated with TRIzol methodology and mRNA was assessed with rat specific **B** BNP, and **C** MPO primers in duplicate. Results were normalized by β-actin mRNA. Plasma was also collected after euthanized, extracted with ethyl acetate, and levels of 14, 15-DHET **D** and 20-HETE **E** were assessed by ELISA assay. Results were expressed as a mean ± standard deviation (SD). *p < 0.05 and **p < 0.01

The results obtained in the cell study were confirmed in female Sprague–Dawley rats using a protocol represented in Fig. 1B. The initial body weights of the rats in the control group (rats that received saline) and DOX group (rats that received DOX) were similar at the beginning of the experiment (~ 242 g BW). Because one of the outcomes of DOX treatment in animals is a reduction in body weight gain as described previously [14], body weights for the rats were measured daily. After the first injection, the saline control group gained body weight faster than the group with DOX treatment (Fig. 4A). At 3 days after the first injection, the body weight and the weight gain of the control group were higher than the DOX group (control group 269 ± 11 g vs. 244 ± 10 g DOX-treated group). In the following days, the difference in body weight and weight gain between the two groups kept increasing, clearly demonstrating that DOX was well administered. At the end of the experimental protocol, the body weight or the weight gain of the control group was ~ 20% higher than the DOX group being 325 ± 17 g vs. 269 ± 15 g (Fig. 4A).

According to Reagan et al. [14], the presence of vacuolization in heart tissue is a sign of cardiotoxicity, which was observed after 4 weeks recovery following DOX injection (3 mg/kg/wk for two weeks). Using the present experimental protocol, no vacuolization was observed at 40X magnification after two weeks recovery after DOX injections (3 mg/kg/wk for two weeks). Thus, histological analyses suggest that cardiotoxicity did not develop after two weeks in our rat model. Levels of BNP mRNA did not change at 48 h after DOX injections when compared to the control group. However, after 2 weeks recovery, levels of BNP and MPO mRNA increased ~ 2.5- and 2-fold, respectively (Fig. 4B and C). Levels of 14,15-DHET in rat plasma samples did not change two days after injection but increased after 2 weeks recovery from DOX injection (Fig. 4D). No changes were observed in 20-HETE levels (Fig. 4E).

**Fig. 5:**
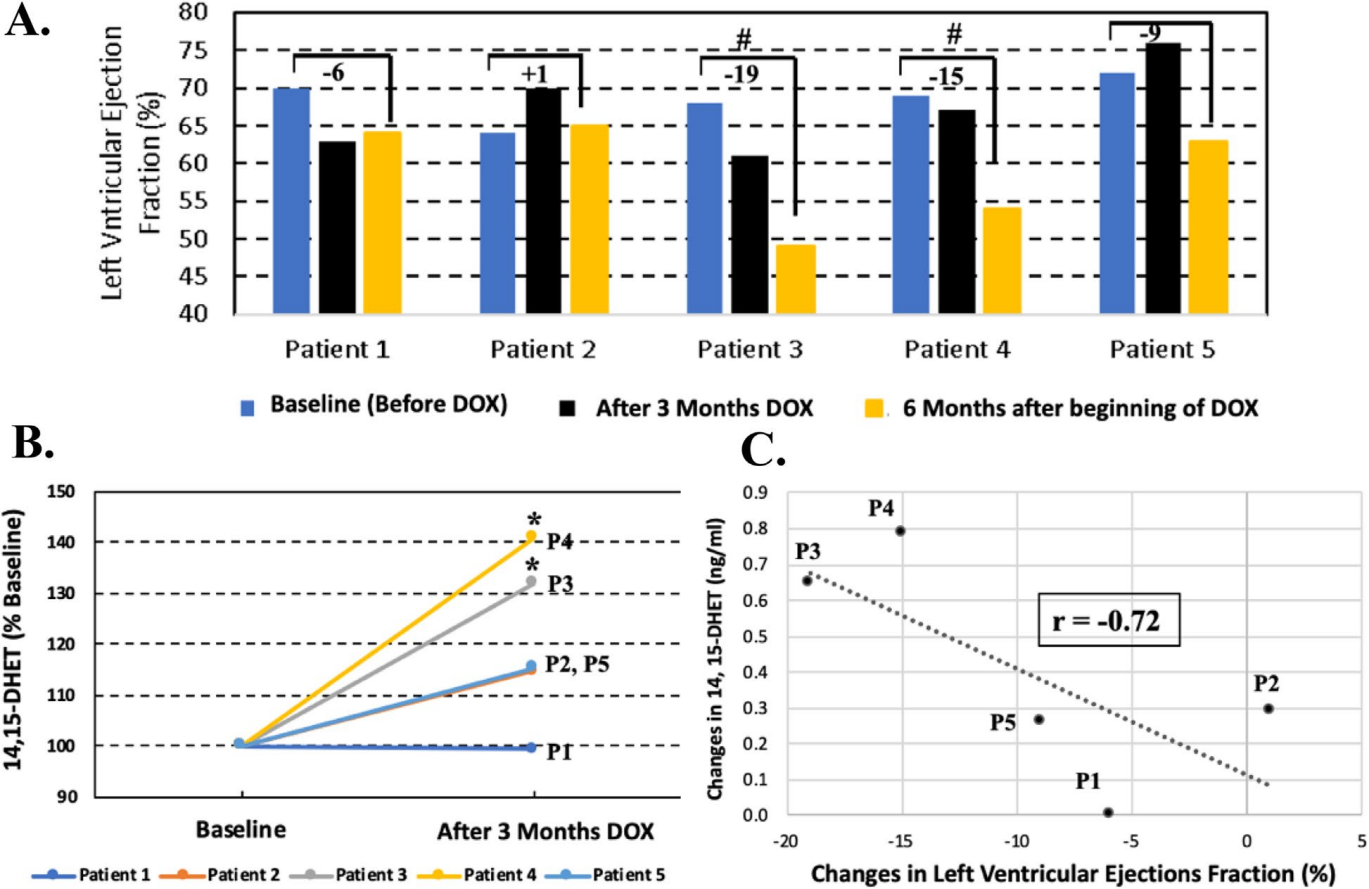
Comparison of changes in 14,15-DHET levels and LVEF of DOX-treated breast cancer patients. **A** Left ventricular ejections fraction (LVEF) measured before, after 3 months anthracycline (DOX) treatment and at 6 months after the beginning of chemotherapy for breast cancer patients. #, reduction of LVEF higher than 10% to < 55% (cardiotoxicity). **B** Increase of 14,15-DHET in plasma samples of breast cancer patients collected before and after 3 months of chemotherapy measured by ELISA. *p < 0.05, triplicate values obtained by ELISA for each patient after chemotherapy compared to before chemotherapy using paired T-test. **C** Relationship of changes in LVEF (%) and changes in 14,15-DHET levels. Correlation coefficient is presented as an r value obtained using Pearson Correlation

14,15-DHET and 20-HETE were measured using plasma samples from breast cancer patients before and after 3 months of DOX treatment (240 mg/m^2^). LVEF was assessed by echocardiography before and three, and six months after the beginning of DOX treatment (Fig. 1C). Patients 3 and 4 developed cardiotoxicity since they exhibited 32% and 41% increased levels of 14,15-DHET at 3 months after beginning of 3 months chemotherapy (after 3 months of chemotherapxy), respectively (*p* < 0.05). The reduction in LVEF in patients 3 and 4 were 19% and 15%, respectively, at 6 months after beginning of 3 months chemotherapy (Fig. 5A and B). The LVEF reductions were higher than 10% with post therapy LVEF values of 49% and 54%, respectively.

**Fig. 6:**
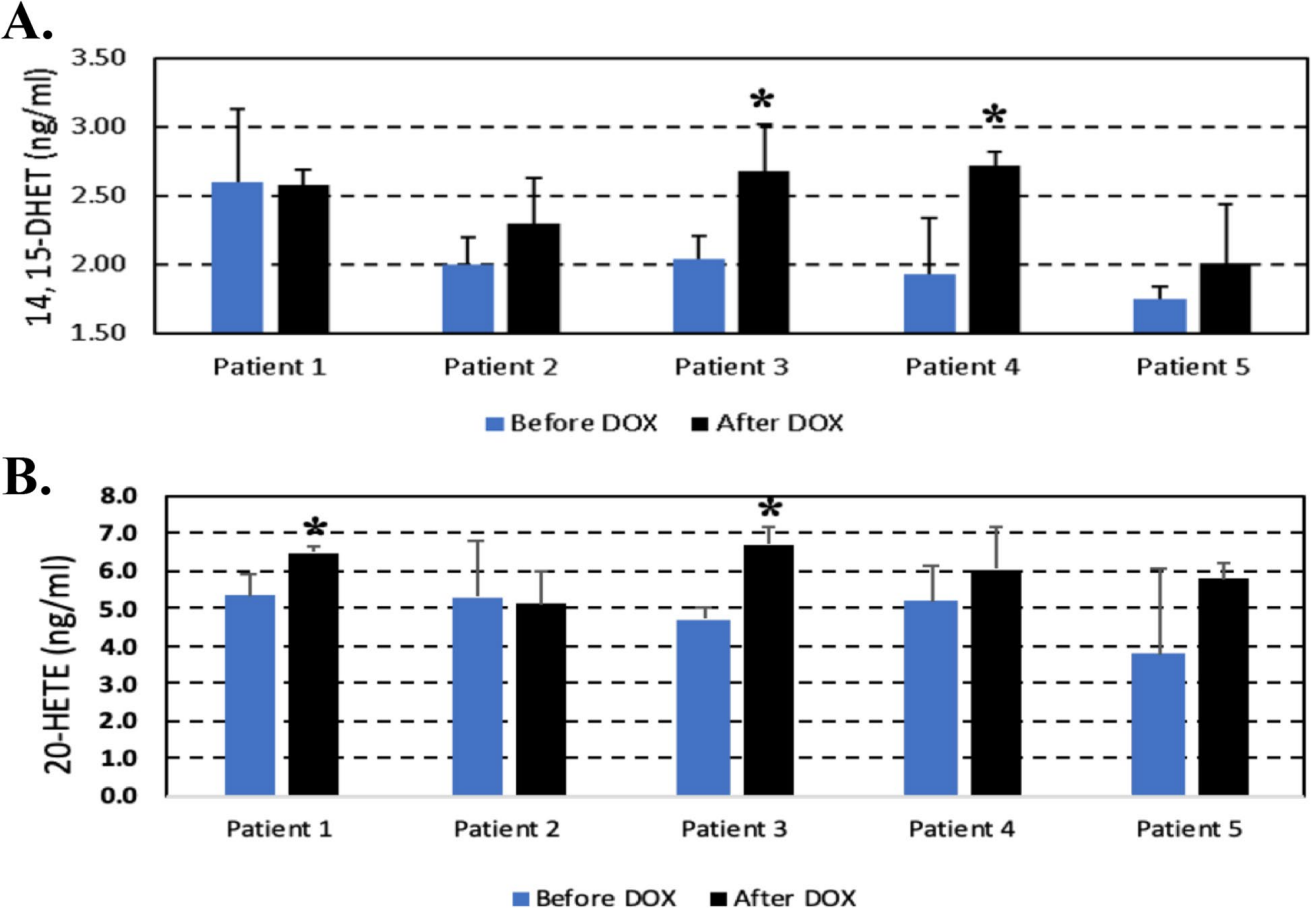
Increased plasma levels of 14,15-DHET (**A**) or 20-HETE (**B**) of DOX-treated breast cancer patients. Plasma samples of breast cancer patients were collected before and after 3 months of chemotherapy. Data are presented as mean ± SD. *p < 0.05, triplicate values obtained by ELISA for each patient after chemotherapy compared to before chemotherapy using paired T-test

Levels of 14,15-DHET did not significantly increase in Patients 1, 2 and 5 after 3 months of chemotherapy compared to their baseline levels (Fig. 6A). Patients 1, 2 and 5 displayed a 6% reduction, 1% increase and 9% reduction of LVEF, respectively, compared with baseline levels (Figs. 5A and 6A). These values of LVEF reduction were lower than 10% are not assigned as patients with CTRCD as predicted by an insignificant increase in 14,15-DHET levels compared to baseline levels at 3 months earlier (Figs. 5A and 6A). In addition, the correlation coefficient between an increase in levels of 14,15-DHET after 3 months of chemotherapy and a decrease in LVEF at 6 months after the beginning of chemotherapy was -0.72 (Fig. 5C). Whereas levels of 14,15-DHET increased 32% and 42% for both Patients 3 and 4, respectively, who developed cardiotoxicity 3 months later, the level of 20-HETE did not increase in plasma of Patient 4. The level of 20-HETE increased in plasma of Patient 1 who did not develop cardiotoxicity (Figs. 5A and 6B).

## Discussion

Early identification of patients susceptible to developing chemotherapy-induced cardiotoxicity could offer opportunities for treatment to avoid or reduce cardiac complications. In this study, we present evidence suggesting that 14,15-DHET might be a predictor of chemotherapy-induced cardiotoxicity. Moreover, the results in cellular and animal model were further supported by human research in breast cancer patients. Cardiomyocytes treated with DOX secreted increased levels of 14,15-DHET to the cell media prior to occurrence of cardiomyocyte hypertrophy, a hallmark of cardiotoxicity. In agreement, levels of 14–15-DHET also increased in rats treated with DOX prior to changes in cardiomyocytes morphology. Finally, levels of 14–15-DHET seem to increase three months prior to decrease in LVEF of DOX-treated cancer patients.

The CYP epoxygenases, CYP2C and CYP2J, metabolize arachidonic acid to 14,15-EET which has cardioprotective, vasodilatory, angiogenic, anti-inflammatory, and analgesic effects [15–19]. The conversion of 14,15-EET to 14,15-DHET by sEH results in a decreased capacity of vasodilation and control of hypertension [15, 16, 19–21]. 14,15-DHET levels increased in plasma and urine samples obtained from patients with abdominal aortic calcification [17], coronary heart disease (CHD) [12], preeclampsia [22] and microvascular angina [23]. Therefore, our results support these findings that an increase in levels of 14,15-DHET in blood is a biomarker for cardiovascular complications and could be used to predict decrease in LVEF of DOX-treated cancer patients.

DOX-induced cardiotoxicity has been reported using the H9c2 cells [11, 24–27]. DOX-induced toxicity, including oxidative stress, cell mortality and apoptosis, were assessed using H9c2 cells by Dallons et al. [27] and it was found that their results agreed with results obtained by other researchers using the same cell type. Due to the challenge to obtain primary neonatal cardiomyocytes, several studies were carried out to compare pharmacological response between rat primary neonatal cardiomyocytes and H9c2 cells and consistent results were found validating the use of H9c2 [28–30]. Thus, the cardiotoxicity assay and cell death inhibitor treatment results obtained using H9c2 cells agreed with the results obtained with rat primary cardiomyocytes cells, which validate the use of H9c2 cells as a model for cardiac hypertrophy. The finding that increased level of 14,15-DHET is an early biomarker of H9c2 cell hypertrophy is verified in a rat model of DOX treatment to show early increase of plasma levels of 14,15-DHET is detected without vacuolization of cardiac tissue.

Hypertrophy of the myocardium is the major cellular alteration underlying a decrease in LVEF following chemotherapy [31]. To verify if 14,15-DHET is secreted by the myocardium and if 14,15-DHET is indeed a biomarker for myocardial hypertrophy induced by DOX, H9c2 cells were treated for 2 h with 1 µM of DOX and cells and cell media were analyzed after different recovery periods. The dose of 1 µM of DOX was selected since peaks of DOX can reach to levels above 1 µM in cancer patients ranging from normal weight to obese [32]. Similar concentration was used previously with the same cell type [24]. In the present study, 2 h incubation of H9c2 cells with DOX (1 µM) increased levels of 14,15-DHET in the cell media prior to increase of cellular area, confirming the use of 14,15-DHET as an early biomarker for DOX-induced cardiomyocyte hypertrophy.

Along with increase of cardiomyocyte size, the elevated BNP mRNA level is a well-accepted marker that increases in myocardial area and cardiotoxicity in in-vitro studies. Two hours after DOX treatment, the BNP mRNA level increased more than 4-fold and the 14,15-DHET level increased in cell media 3-fold compared to untreated cells. There was minimal change in cellular viability as previously reported for the same recovery time [8]. Alsaad et al. [25] reported that 1 µM of DOX treatment for 6 h increased the BNP mRNA level in H9c2 cells. It was reported in a rat study that 6 doses of DOX (in total, 15 mg/kg) administered in 2 weeks increased hypertrophy, assessed by increase in ANP mRNA level and increased sEH mRNA expression in heart tissue and sEH activity (calculated by 14,15-DHET/14,15-EET) in heart microsomes after 14 days of recovery [25]. Gene expression of CYP epoxygenases, CYP2C and CYP2J which convert arachidonic acid to EETs, were not upregulated at the same time point in heart tissue. The 14,15-DHET level increased in heart tissue but 14,15-EET-formation activity or total epoxygenase activity (14,15-DHET + 14,15-EET) level in heart microsomes did not increase [25]. In the present study, 1 µM of DOX treatment for 2 h increased BNP mRNA in H9c2 cells and 14,15-DHET levels in media increased when no change in cell area was noted at 2 h after treatment. Treatment of DOX for two weeks (in total, 6 mg/kg) increased BNP mRNA levels after two weeks of recovery with a concomitant increase in 14,15-DHET levels in blood without vacuolization of rat cardiac tissue. Therefore, our results indicate that DOX treatment increases sEH (activity and gene expression) in cardiomyocytes, which further increase the 14,15-DHET level in heart tissue and blood prior to onset of cardiomyocyte hypertrophy. This effect can be easily monitored by a blood test.

Increased levels of myeloperoxidase (MPO), which catalyzes the formation of a number of reactive oxidant species, is linked with cardiovascular complications [33]. Using plasma of breast cancer patients treated with anthracycline, Ky et al. [3] have suggested that the increase in MPO level provides an early information on the risk of developing cardiotoxicity. Here our novel findings are that the MPO mRNA level in rat heart tissue increased after treatment of rats with DOX without vacuolization of cardiac tissue as plasma 14,15-DHET level increased prior to any DOX-induced heart tissue damage.

Chemotherapy-associated CTRCD is defined as either a cardiomyopathy with a reduction of LVEF ≥ 5% to < 55% and with symptoms of heart failure or as asymptomatic with a reduction of LVEF ≥ 10% to < 55% [9]. Once this CTRCD condition is identified, the window of opportunity for treatment might be too short. In this study, levels of 14,15-DHET in plasma increased three months before a decrease in LVEF was detected in breast cancer patients treated with a chemotherapeutic agent (DOX). Our findings from basic research using in-vitro and in-vivo approach, were consistent with data from five breast cancer patients before and after their 3 months clinical treatment with DOX. The increased plasma 14,15-DHET level was closely associated with decreased left ventricle ejection fraction (% LVEF) measured by pre- and post-chemotherapy echocardiography. While a pilot due to human sample size, this study provides proof-of-principle that a new class of biomarker, bioactive lipid, should be studied as a biomarker in cardio-oncology.

Since a large number of researchers have suggested that increased conversion of EET to DHET by sEH is linked to several cardiovascular complications, inhibition of sEH was tested as a therapeutical approach towards these complications [21, 26]. Pharmacological inhibition of sEH activity was effectively used to treat heart failure in animal studies [26]. Moreover, genetical inhibition of sEH expression improved post ischemic functional recovery of mice [34]. Based on successful animal studies, clinical trials have been carried out using sEH inhibitors, e.g., AR9281 [21, 35]. Moreover, it was shown that two inhibitors of sEH, could decrease markers of hypertrophy when those were used prior to (24 h) and during 1 µM of 6 h DOX treatment of H9c2 cells [25]. Thus, inhibition of sEH may represent a therapeutic approach to manage human cardiovascular complications such as DOX-induced cardiotoxicity and the effect of this inhibition may be monitored by assessing 14,15-DHET in plasma of patients.

Cardioprotective activity of dexrazoxane (DEX) against DOX-induced cardiotoxicity has been demonstrated in basic research and clinical studies [36, 37]. Dallons et al. [27] reported the main features of DOX-induced cell toxicity such as oxidative stress and antioxidant activity of DEX in H9c2 cells. Since the results from this translational study convincingly indicate that sEH activity increased after DOX treatment, adding an sEH inhibitor may be beneficial against DOX-induced cardiotoxicity as the mechanism of action of the sEH inhibitor (vasodilation) differs from antioxidant activity of DEX. Nevertheless, the benefits of sEH inhibition in DOX-treated cancer patients should be carefully evaluated, since a group of researchers demonstrated that levels of 14,15-EET increased in breast cancer tissues compared to adjacent noncancerous tissue and that overexpression of sEH attenuated proliferation and migration in human breast cancer cells [38]. Moreover, Allison et al. [39] demonstrated that overexpression of CYP2J diminished effect of anthracyclines in breast cancer cells, such as the effect of this drug on cancer cell apoptosis and cell viability, which indicated that increased 14,15-EET level would increase the aggressiveness of the tumor [39]. However, findings from the same articles show that an antioxidant enzyme, aldehyde dehydrogenase (ALDH), mediates 14,15-EET effect on cancer cells and that silencing ALDH activity would improve the effect of anthracycline [39]. Therefore, inhibition of ALDH along with sEH in DOX-treated patients could eliminate the effect of 14,15-EET on cancer cells and allow antihypertensive property of 14,15-EET to protect heart tissue against DOX-induced cardiotoxicity. However, the therapeutical strategy should be carefully tested in further studies.

The increased level of 14,15-DHET [12, 22] or 20-HETE [40] (bioactive lipid) was implicated in hypertension and CHD. However, in this study, after treatment of rats with DOX, only plasma levels of 14,15-DHET (sEH metabolite) increased, not the levels of 20-HETE (CYP 4A/4F metabolite). The results demonstrated that inflammation is not the only mechanism for the early increase of sEH expression after DOX treatment. This study provides a new class of early biomarker, bioactive lipid, which should be studied as a biomarker in cardio-oncology.

In conclusion, the results from our research using cell and animal models seem to be translatable to breast cancer patients indicating that levels of 14,15-DHET are elevated prior to major changes in cardiac function after exposure to chemotherapy agents. The results of this hypothesis generating study indicate that increased levels of 14,15-DHET in plasma could be an important clinical biomarker. However further research with a larger sample size is needed to understand its clinical utility. Identification of vulnerable patients who develop chemotherapy-induced cardiotoxicity by measurement of 14,15-DHET levels could also lead to a novel strategy for treatment of cardiac complications in cancer patients.

## Data Availability

The authors can confirm that all relevant data are included in the article.

## Abbreviations

DOX: Doxorubicin
LVEF: Left ventricular ejection fraction
2D STE: Two-dimensional speckle tracking echocardiography
LVGLS: Left ventricular global longitudinal strain
TN: Troponin
BNP: Brain natriuretic peptide
miRNA: MicroRNA
14,15-DHET: 14,15-Dihydroxyeicosatrienoic acid
20-HETE: 20-Hydroxyeicosatetraenoic acid
sEH: Soluble epoxide hydrolase
CYP: Cytochrome P450
EET: Epoxyeicosatrienoic acid
MTT: 3-(4,5-Dimethylthiazol-2-yl)-2,5-diphenyl tetrazolium bromide
DMEM: Dulbecco’s modified Eagle’s medium
EDTA: Ethylenediaminetetraacetic acid
MPO: Myeloperoxidase
CTRCD: Cancer therapy-related cardiac dysfunction
CHD: Coronary heart disease
ANP: Atrial natriuretic peptide
EODB: 3-[2-(4-Ethylphenyl)-2-oxoethyl]-1,2-dimethyl-1H-3,1-benzimidazol-3-Iumbromide
ALDH: Aldehyde dehydrogenase

## References

[1] Siegel RL, Miller KD, Wagle NS, Jemal A (2023). Cancer statistics, 2023. CA Cancer J Clin.

[2] Thorn CF, Oshiro C, Marsh S, Hernandez-Boussard T, McLeod H, Klein TE, Altman RB (2011). Doxorubicin pathways: pharmacodynamics and adverse effects. Pharmacogenet Genomics.

[3] Ky B, Putt M, Sawaya H, French B, Januzzi JL, Sebag IA, Plana JC, Cohen V, Banchs J, Carver JR, Wiegers SE, Martin RP, Picard MH, Gerszten RE, Halpern EF, Passeri J, Kuter I, Scherrer-Crosbie M (2014). Early increases in multiple biomarkers predict subsequent cardiotoxicity in patients with breast cancer treated with doxorubicin, taxanes, and trastuzumab. J Am Coll Cardiol.

[4] Ribeiro ML, Jorge AJL, Nacif MS, Martins WA (2019). Early detection and monitoring of cancer chemotherapy-related left ventricular dysfunction by imaging methods. Ar Bras Cardiol.

[5] Gent DG, Rebecca D (2023). The 2022 European Society of Cardiology Cardio-oncology Guidelines in Focus. Eur Cardiol.

[6] Brown C, Mantzaris M, Nicolaou E, Karanasiou G, Papageorgiou E, Curigliano G, Cardinale D, Filippatos G, Memos N, Naka KK, Papakostantinou A, Vogazianos P, Ioulianou E, Shammas C, Constantinidou A, Tozzi F, Fotiadis DI, Antoniades A (2022). A systematic review of miRNAs as biomarkers for chemotherapy-induced cardiotoxicity in breast cancer patients reveals potentially clinically informative panels as well as key challenges in miRNA research. Cardiooncology.

[7] Bär C, Chatterjee S, FalcãoPires I, Rodrigues P, Sluijter JPG, Boon RA, Nevado RM, Andrés V, Sansonetti M, de Windt L, Ciccarelli M, Hamdani N, Heymans S, FiguinhaVideira R, Tocchetti CG, Giacca M, Zacchigna S, Engelhardt S, Dimmeler S, Madonna R (2020). Non-coding RNAs: update on mechanisms and therapeutic targets from the ESC Working Groups of Myocardial Function and Cellular Biology of the Heart. Cardiovasc Res.

[8] Zordoky BN, Anwar-Mohamed A, Aboutabl ME, El-Kadi AO (2010). Acute doxorubicin cardiotoxicity alters cardiac cytochrome P450 expression and arachidonic acid metabolism in rats. Toxicol Appl Pharmacol.

[9] Seidman A, Hudis C, Pierri MK, Shak S, Paton V, Ashby M, Murphy M, Stewart SJ, Keefe D (2002). Cardiac dysfunction in the trastuzumab clinical trials experience. J Clin Oncol.

[10] Lang RM, Bierig M, Devereux RB, Flachskampf FA, Foster E, Pellikka PA, Picard MH, Roman MJ, Seward J, Shanewise JS, Solomon SD, Spencer KT, Sutton MS, Stewart WJ, Chamber Quantification Writing Group, American Society of Echocardiography’s Guidelines and Standards Committee, European Association of Echocardiography (2005). Recommendations for chamber quantification: a report from the American Society of Echocardiography’s Guidelines and Standards Committee and the Chamber Quantification Writing Group, developed in conjunction with the European Association of Echocardiography, a branch of the European Society of Cardiology. J Am Soc Echocardiogr..

[11] Tse MM, Aboutabl ME, Althurwi HN, Elshenawy OH, Abdelhamid G, El-Kadi AO (2013). Cytochrome P450 epoxygenase metabolite, 14,15-EET, protects against isoproterenol-induced cellular hypertrophy in H9c2 rat cell line. Vascul Pharmacol.

[12] Yang T, Peng R, Guo Y, Shen L, Zhao S, Xu D (2013). The role of 14,15-dihydroxyeicosatrienoic acid levels in inflammation and its relationship to lipoproteins. Lipids Health Dis.

[13] Benite-Ribeiro SA, Lucas-Lima KL, dos Santos JM, Cagnini DQ, Guimarães IG (2021). Early type 2 diabetes and obesity does not affect eicosanoids level and renal morphology in a rat model. Braz J Dev.

[14] Reagan WJ, York M, Berridge B, Schultze E, Walker D, Pettit S (2013). Comparison of cardiac troponin I and T, including the evaluation of an ultrasensitive assay, as indicators of doxorubicin-induced cardiotoxicity. Toxicol Pathol.

[15] Capdevila JH, Wang W, Falck JR (2015). Arachidonic acid monooxygenase: genetic and biochemical approaches to physiological/pathophysiological relevance. Prostaglandins Other Lipid Mediat.

[16] Xiao B, Li X, Yan J, Yu X, Yang G, Xiao X, Voltz JW, Zeldin DC, Wang DW (2010). Overexpression of cytochrome P450 epoxygenases prevents development of hypertension in spontaneously hypertensive rats by enhancing atrial natriuretic peptide. J Pharmacol Exp Ther.

[17] Inceoglu B, Jinks SL, Ulu A, Hegedus CM, Georgi K, Schmelzer KR, Wagner K, Jones PD, Morisseau C, Hammock BD (2008). Soluble epoxide hydrolase and epoxyeicosatrienoic acids modulate two distinct analgesic pathways. Proc Natl Acad Sci U S A.

[18] Liu P, Zhang S, Gao J, Lin Y, Shi G, He W, Touyz RM, Yan L, Huang H (2018). Downregulated serum 14, 15-epoxyeicosatrienoic acid is associated with abdominal aortic calcification in patients with primary aldosteronism. Hypertension.

[19] Gauthier KM, Edwards EM, Falck JR, Reddy DS, Campbell WB (2005). 14,15-epoxyeicosatrienoic acid represents a transferable endothelium-dependent relaxing factor in bovine coronary arteries. Hypertension.

[20] Lin WK, Falck JR, Wong PY (1990). Effect of 14,15-epoxyeicosatrienoic acid infusion on blood pressure in normal and hypertensive rats. Biochem Biophys Res Commun.

[21] Imig JD, Hammock BD (2009). Soluble epoxide hydrolase as a therapeutic target for cardiovascular diseases. Nat Rev Drug Discov.

[22] Santos JM, Park JA, Joiakim A, Putt DA, Taylor RN, Kim H (2017). The role of soluble epoxide hydrolase in preeclampsia. Med Hypotheses.

[23] Akasaka T, Sueta D, Arima Y, Tabata N, Takashio S, Izumiya Y, Yamamoto E, Yamamuro M, Tsujita K, Kojima S, Kaikita K, Kajiwara A, Morita K, Oniki K, Saruwatari J, Nakagawa K, Ogata Y, Matsui K, Hokimoto S (2016). Association of CYP2C19 variants and epoxyeicosatrienoic acids on patients with microvascular angina. Am J Physiol Heart Circ Physiol.

[24] Pereira-Oliveira M, Reis-Mendes A, Carvalho F, Remião F, Bastos ML, Costa VM (2019). Doxorubicin is key for the cardiotoxicity of FAC (5-Fluorouracil + Adriamycin + Cyclophosphamide) combination in differentiated H9c2 cells. Biomolecules.

[25] Alsaad AM, Zordoky BN, El-Sherbeni AA, El-Kadi AO (2012). Chronic doxorubicin cardiotoxicity modulates cardiac cytochrome P450-mediated arachidonic acid metabolism in rats. Drug Metab Dispos.

[26] Qiu H, Li N, Liu JY, Harris TR, Hammock BD, Chiamvimonvat N (2011). Soluble epoxide hydrolase inhibitors and heart failure. Cardiovasc Ther.

[27] Dallons M, Schepkens C, Dupuis A, Tagliatti V, Colet JM (2020). New insights about doxorubicin-induced toxicity to cardiomyoblast-derived H9C2 cells and dexrazoxane cytoprotective effect: contribution of in vitro^1^H-NMR metabonomics. Front Pharmacol.

[28] Watkins SJ, Borthwick GM, Arthur HM (2011). The H9C2 cell line and primary neonatal cardiomyocyte cells show similar hypertrophic responses in vitro. In Vitro Cell Dev Biol Anim.

[29] Gergely S, Hegedűs C, Lakatos P, Kovács K, Gáspár R, Csont T, Virág L (2015). High throughput screening identifies a novel compound protecting cardiomyocytes from doxorubicin-induced damage. Oxid Med Cell Longev.

[30] Gasser A, Chen YW, Audebrand A, Daglayan A, Charavin M, Escoubet B, Karpov P, Tetko I, Chan MWY, Cardinale D, Désaubry L, Nebigil CG (2019). Prokineticin receptor-1 signaling inhibits dose- and time-dependent anthracycline-Induced cardiovascular toxicity via myocardial and vascular protection. JACC CardioOncol.

[31] Florescu M, Cinteza M, Vinereanu D (2013). Chemotherapy-induced cardiotoxicity. Maedica (Bucur).

[32] Barpe DR, Rosa DD, Froehlich PE (2010). Pharmacokinetic evaluation of doxorubicin plasma levels in normal and overweight patients with breast cancer and simulation of dose adjustment by different indexes of body mass. Eur J Pharm Sci.

[33] Ndrepepa G (2019). Myeloperoxidase - a bridge linking inflammation and oxidative stress with cardiovascular disease. Clin Chim Acta.

[34] Edin ML, Hamedani BG, Gruzdev A, Graves JP, Lih FB, Arbes SJ, Singh R, Orjuela Leon AC, Bradbury JA, DeGraff LM, Hoopes SL, Arand M, Zeldin DC (2018). Epoxide hydrolase 1 (EPHX1) hydrolyzes epoxyeicosanoids and impairs cardiac recovery after ischemia. J Biol Chem.

[35] Chen D, Whitcomb R, MacIntyre E, Tran V, Do ZN, Sabry J, Patel DV, Anandan SK, Gless R, Webb HK (2012). Pharmacokinetics and pharmacodynamics of AR9281, an inhibitor of soluble epoxide hydrolase, in single- and multiple-dose studies in healthy human subjects. J Clin Pharmacol.

[36] Galetta F, Franzoni F, Cervetti G, Cecconi N, Carpi A, Petrini M, Santoro G (2005). Effect of epirubicin-based chemotherapy and dexrazoxane supplementation on QT dispersion in non-Hodgkin lymphoma patients. Biomed Pharmacother.

[37] Ducroq J, MohaouMaati H, Guilbot S, Dilly S, Laemmel E, Pons-Himbert C, Faivre JF, Bois P, Stücker O, Le Grand M (2010). Dexrazoxane protects the heart from acute doxorubicin-induced QT prolongation: a key role for I(Ks). Br J Pharmacol.

[38] Wei X, Zhang D, Dou X, Niu N, Huang W, Bai J, Zhang G (2014). Elevated 14,15- epoxyeicosatrienoic acid by increasing of cytochrome P450 2C8, 2C9 and 2J2 and decreasing of soluble epoxide hydrolase associated with aggressiveness of human breast cancer. BMC Cancer.

[39] Allison SE, Chen Y, Petrovic N, Zhang J, Bourget K, Mackenzie PI, Murray M (2017). Activation of ALDH1A1 in MDA-MB-468 breast cancer cells that over-express CYP2J2 protects against paclitaxel-dependent cell death mediated by reactive oxygen species. Biochem Pharmacol.

[40] Hoopes SL, Garcia V, Edin ML, Schwartzman ML, Zeldin DC (2015). Vascular actions of 20-HETE. Prostaglandins Other Lipid Mediat.

